# Spatiotemporal Variation in Aboveground Biomass and Its Response to Climate Change in the Marsh of Sanjiang Plain

**DOI:** 10.3389/fpls.2022.920086

**Published:** 2022-06-21

**Authors:** Yiwen Liu, Xiangjin Shen, Yanji Wang, Jiaqi Zhang, Rong Ma, Xianguo Lu, Ming Jiang

**Affiliations:** ^1^Northeast Institute of Geography and Agroecology, Chinese Academy of Sciences, Changchun, China; ^2^University of Chinese Academy of Sciences, Beijing, China; ^3^College of Mapping and Geographical Sciences, Liaoning Technical University, Fuxin, China

**Keywords:** marsh wetland, biomass, NDVI, climatic change, Sanjiang Plain

## Abstract

The Sanjiang Plain has the greatest concentration of freshwater marshes in China. Marshes in this area play a key role in adjusting the regional carbon cycle. As an important quality parameter of marsh ecosystems, vegetation aboveground biomass (AGB) is an important index for evaluating carbon stocks and carbon sequestration function. Due to a lack of *in situ* and long-term AGB records, the temporal and spatial changes in AGB and their contributing factors in the marsh of Sanjiang Plain remain unclear. Based on the measured AGB, normalized difference vegetation index (NDVI), and climate data, this study investigated the spatiotemporal changes in marsh AGB and the effects of climate variation on marsh AGB in the Sanjiang Plain from 2000 to 2020. Results showed that the marsh AGB density and annual maximum NDVI (NDVI_max_) had a strong correlation, and the AGB density could be accurately calculated from a power function equation between NDVI_max_ and AGB density (AGB density = 643.57 × NDVI${}_{\text{max}}^{4.2474}$). According to the function equation, we found that the AGB density significantly increased at a rate of 2.47 g·C/m^2^/a during 2000–2020 in marshes of Sanjiang Plain, with the long-term average AGB density of about 282.05 g·C/m^2^. Spatially, the largest increasing trends of AGB were located in the north of the Sanjiang Plain, and decreasing trends were mainly found in the southeast of the study area. Regarding climate impacts, the increase in precipitation in winter could decrease the marsh AGB, and increased temperatures in July contributed to the increase in the marsh AGB in the Sanjiang Plain. This study demonstrated an effective approach for accurately estimating the marsh AGB in the Sanjiang Plain using ground-measured AGB and NDVI data. Moreover, our results highlight the importance of including monthly climate properties in modeling AGB in the marshes of the Sanjiang Plain.

## Introduction

Wetlands are one of the world's major ecosystems that play an important role in the global carbon cycle and ecological equilibrium (Hu et al., 2017; Liu et al., 2021). Marsh is a type of wetland ecosystem, which is crucial for regulating the regional climate and carbon cycle (Shen et al., 2020; Rietl et al., 2021; Wang et al., 2022a). As an important quality parameter of marsh ecosystems, vegetation aboveground biomass (AGB) is an important index for evaluating carbon stocks and carbon sequestration function in marshes (Shen et al., 2021a; Wang et al., 2021). Since climatic change remarkably influences marsh vegetation, understanding the impact of climatic change on marsh AGB is important for assessing the carbon storage of wetland ecosystems in the context of global climate change. Many studies have analyzed the spatiotemporal variations of vegetation AGB and their responses to climate change (Stegen et al., 2011; Shi et al., 2015; Castanho et al., 2020; Zhou et al., 2021). However, most of these studies focused on grassland and forest ecosystems, and only a few were conducted on marsh ecosystems (Flannigan et al., 2000; Shen et al., 2016, 2022a; Dai et al., 2021; Konings et al., 2021; Naik et al., 2021; Nandy et al., 2021; Qin et al., 2021; Wang et al., 2022b). Compared with other ecosystems, marsh ecosystems have unique environmental conditions, which may lead to different influences of climate change on AGB (Wang et al., 2021). In the context of climate change, clarifying the temporal and spatial variations of marsh AGB is very important for predicting the regional carbon cycle.

The Sanjiang Plain has the largest freshwater marsh in China (Wang et al., 2006). The marsh on the Sanjiang Plain plays a crucial role in regional biodiversity conservation and the carbon cycle (Sui et al., 2019). Some studies have analyzed the variations in marsh AGB and the effects of climatic change on marsh AGB in the Sanjiang Plain (Shi et al., 2015; Li et al., 2021). For example, Ni et al. (1996) studied the influences of hydrothermal conditions and the AGB of *Deyeuxia angustifolia* in the Sanjiang Plain marshes. Shi et al. (2015) analyzed the effects of different water levels on the AGB of vegetation in the freshwater marshes of the Sanjiang Plain and found that increasing water levels promoted the biomass of *D. angustifoli*a. Li et al. (2021) analyzed the influences of temperature and precipitation on AGB and found that the increase of precipitation can increase the AGB in the Sanjiang Plain. However, these studies focused on analyzing changes in marsh AGB and their responses to climatic change at a species scale, and no research has been conducted over the entire Sanjiang Plain due to the limitations of field investigations. Climate change could induce different responses of different marsh vegetation in a region or the same marsh vegetation in different regions to climate changes (Shen et al., 2019a, 2021b). Thus, studying the variations in marsh AGB over the entire Sanjiang Plain and the influences of climate change on marsh AGB is necessary. Compared with measured sampling, remote sensing data have the advantage of comprehensive spatiotemporal coverage (Ozesmi and Bauer, 2002; Gerber et al., 2018; Yang et al., 2021; Shen et al., 2022a,b). Owing to the rapid development of remote sensing technology, many studies have used satellite remote sensing datasets combined with measured ground AGB datasets to estimate the vegetation AGB. Yang et al. (2018) established an effective model in the three-river headwaters region to estimate grassland AGB by combining the AGB field measurement and remote sensing data. Lumbierres et al. (2017) used the NDVI and measured AGB data to estimate the grassland AGB in three-river headwaters. Piao et al. (2004) suggested that a power function between the annual NDVI_max_ and AGB density could be established to accurately estimate the AGB of grassland in China. Wang et al. (2021) have also constructed a power function to estimate AGB in the Tibetan Plateau marshes. Combined with ground-measured data, remote sensing is a useful tool for long-term monitoring of the AGB of ecosystems (Lumbierres et al., 2017). Therefore, it is necessary to analyze the change in AGB in the Sanjiang Plain by using a combination of remote sensing and measured data. In addition, Wang et al. (2021) reported that daytime and nighttime temperatures had an asymmetric impact on marsh AGB on the Tibetan Plateau. The positive impact of warming the minimum temperature at night was more significant. Compared with the Tibetan Plateau, the Sanjiang Plain has a lower altitude and more precipitation (Zhang et al., 2015). However, whether there is an asymmetric influence of warming daytime and nighttime temperatures on AGB in the Sanjiang Plain marshes remains unclear. Under asymmetric global day and night warming, exploring the effects of the maximum temperature in the daytime and the minimum temperature at night on the AGB in the Sanjiang Plain marshes is necessary.

In this study, an estimation model of marsh AGB was established by using measured biomass data and NDVI in the marshes of the Sanjiang Plain. Based on climate and NDVI datasets during 2000–2020, we investigated the spatiotemporal variation in marsh AGB and the influences of climate factors on marsh AGB. This study aimed to clarify the spatial and temporal changes in AGB and the impacts of climatic change on marsh AGB in the Sanjiang Plain. We put forward two hypotheses: (1) the AGB density of marsh vegetation can be estimated by the NDVI_max_ in the Sanjiang Plain; (2) temperature is the main reason affecting the AGB in the marsh of the Sanjiang Plain. The results of this study can aid in protecting marsh vegetation and predicting the carbon cycle in the Sanjiang Plain marshes. As the marsh in the Sanjiang Plain is a typical temperate freshwater marsh, the findings of this study have important implications for the studies of freshwater marsh in temperate regions.

## Materials and Methods

### Study Area

**Figure 1:**
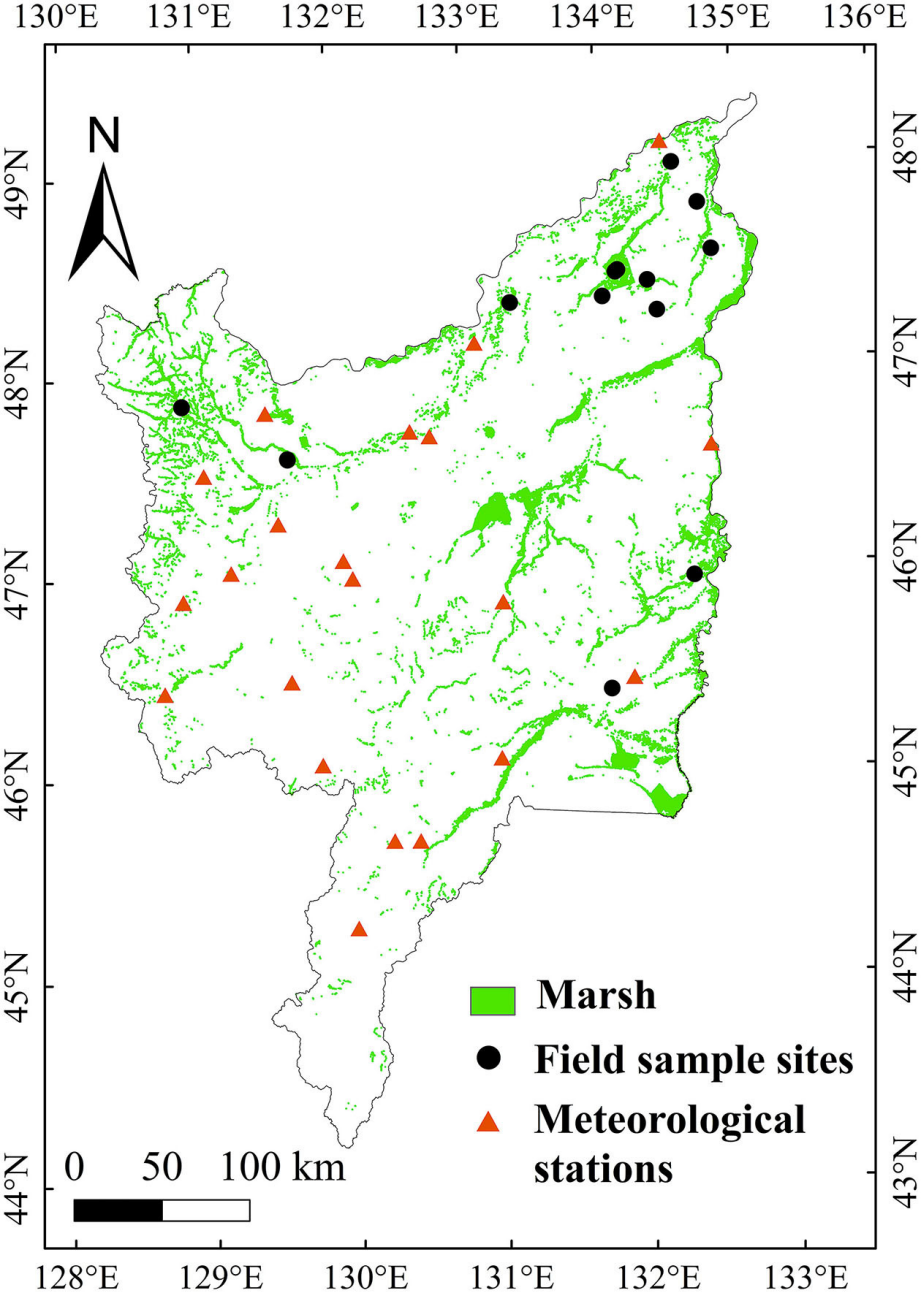
Distributions of the marsh, meteorological stations, and sample sites of aboveground biomass (ABG) at the Sanjiang Plain.

The Sanjiang Plain is located in the northeast of China, with a latitude of 43°49'N to 48°27'N and a longitude of 129°11'E to 135°05'E (Figure 1) (Luo et al., 2022). Sanjiang Plain has a low and flat terrain and is the largest marsh region in China (Mao et al., 2015). The Sanjiang Plain has a continental monsoonal climate, with more than half of the annual precipitation in July and August. The mean annual temperature is approximately 3.2°C, with January and July being the coldest and hottest months, respectively (Wu et al., 2021).

### Data

The climate data used in this study were the monthly minimum temperature (T_min_), maximum temperature (T_max_), mean temperature (T_mean_), and precipitation data from 21 meteorological stations in the Sanjiang Plain during 2000–2020, obtained from the Chinese Meteorological Center (http://www.nmic.cn/). The marsh distribution data were the spatial distribution dataset of China's marsh wetlands in 2000 and 2015, which have been subjected to strict data quality control and verification (Mao et al., 2020). The spatial resolution of marsh distribution data was 30 m (Wang et al., 2015; Mao et al., 2019). The MODIS NDVI data used in this study were from the Goddard Space Flight Center's MOD13Q1 dataset from 2000 to 2020 (https://ladsweb.modaps.eosdis.nasa.gov/). The time resolution of MOD13Q1 NDVI was 16 days, and the spatial resolution was 250 m (Beck et al., 2006). The AGB data were field-observed marsh AGB data collected from 16 survey sites during 2013–2017 in the Sanjiang Plain (Shen et al., 2021a). The collection time of field biomass sampling sites was from July to September. Each sampling site was set up with three repeated quadrats of equal size (1 m × 1 m). To accurately reflect the AGB, the average value of three repeated quadrats was considered the sampling point's value based on the longitude and latitude of the sampling point (Wang et al., 2021). In this study, the marsh vegetation AGB (g) was converted to carbon (g·C) by using a conversion coefficient of 0.45 (Fang et al., 2007; Shen et al., 2021a; Wang et al., 2021).

### Methods

#### Estimation of AGB

According to the marsh distribution data in 2000 and 2015, we extracted the unchanged distribution of marshes in the Sanjiang Plain (marshes both in 2000 and 2015), to eliminate the interference of human activities on marsh wetlands (Shen et al., 2019b). In this study, we converted the 16-day NDVI into annual NDVI_max_ data using the maximum value compositing method (Holben, 1986). The corresponding NDVI_max_ of each sample site was obtained according to geographical position (Wang et al., 2021). We then approximated the AGB using the measured NDVI_max_ and AGB density in the Sanjiang Plain marsh from 2000 to 2020. In this study, the regional averages of the variables are the averages of the corresponding pixels in the Sanjiang Plain marshes (Shen et al., 2016).

#### Model Validation

This study evaluated the equation based on the predicted root mean square error (RMSE) and relative error (Equations 1, 2) (Wang et al., 2021). The symbol *n* denotes the number of effective samples (*n* = 16 in this study).


(1) $$\begin{array}{rcl} RMSE & = & \sqrt{\frac{\overset{n}{\underset{i=1}{\sum }}{\left(y_{estimation}-y_{observation}\right)}^{2}}{n}} \end{array}$$

(2) $$\begin{array}{rcl} Relative\;error\left(\%\right) & = & 100\%\times \frac{|y_{observation}-y_{estimation}|}{y_{observation}} \end{array}$$

#### Trend Analysis

In this study, we used the trend analysis method to calculate the trends of AGB and meteorological factors in the marshes of the Sanjiang Plain. The formula is included below (Shen et al., 2021b):


(3) $$\begin{array}{r} \theta_{\text{slope}}\text{=}\frac{\left(n\times \overset{n}{\underset{i=1}{\sum }}i\times AGB_{i}\right)-\left(\overset{n}{\underset{i=1}{\sum }}i\overset{n}{\underset{i=1}{\sum }}AGB_{i}\right)}{n\times \overset{n}{\underset{i=1}{\sum }}i^{2}-{\left(\overset{n}{\underset{i=1}{\sum }}i\right)}^{2}} \end{array}$$

In Formula (3), *AGB*_*i*_ is the value of AGB or the annual (or monthly) meteorological factor of *year i*, where, *n* represents the length of the study period, i.e., 21 years, *i* is the year serial number, and θ_*slope*_ is the slope of the meteorological factor or AGB change trend of each pixel. If θ_*slope*_ < 0, the change in AGB or meteorological factors in the growing season (or month) of the pixel is a decreasing trend; otherwise, it is an increasing trend.

#### Correlation Analysis

In this study, we interpolated the meteorological data using the Kriging interpolation method, and the grid images of precipitation, T_min_, T_max_, and T_mean_ in the annual (or monthly) of marsh vegetation in the Sanjiang Plain with the same spatial resolution (250 m) as the AGB image were obtained (Shen et al., 2021b). The correlation between meteorological factors in the annual vegetation (or month) and AGB was calculated by using the correlation analysis method. The formula is as follows:


(4) $$\begin{array}{r} R_{xy}=\frac{\overset{n}{\underset{a=1}{\sum }}\left(x_{a}-\bar{x}\right)\left(y_{a}-\bar{y}\right)}{\sqrt{\overset{n}{\underset{a=1}{\sum }}{\left(x_{a}-\bar{x}\right)}^{2}}\sqrt{\overset{n}{\underset{a=1}{\sum }}{\left(y_{a}-\bar{y}\right)}^{2}}} \end{array}$$

In Formula (4), *R*_*xy*_ represents the correlation coefficient, *n* is the length of the study period, *n* = 21 years in this study, *x*_*a*_ is the annual (or monthly) average value of the meteorological factor in a certain year, $\overset{\bar{}}{x}$ is the multi-year average value of the annual (or monthly) average value of the meteorological factor in the past 21 years, *y*_*a*_ is the AGB value of a year, and $\overset{\bar{}}{y}$ is the average value of the multi-year average AGB.

## Results

### The Estimation of Marsh AGB and Its Verification in the Sanjiang Plain

**Figure 2:**
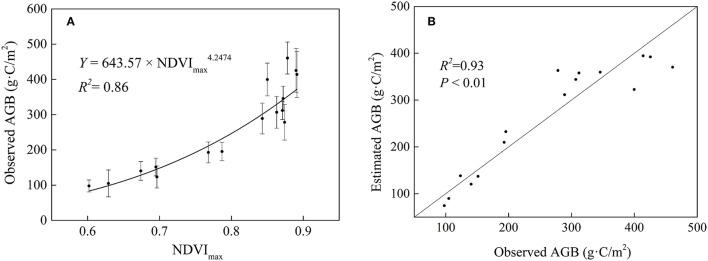
Equation between maximum normalized difference vegetation index (NDVI_max_) and observed AGB density **(A)**, and comparison between estimated AGB density and observed AGB density **(B)** in the marsh of the Sanjiang Plain.

**Figure 3:**
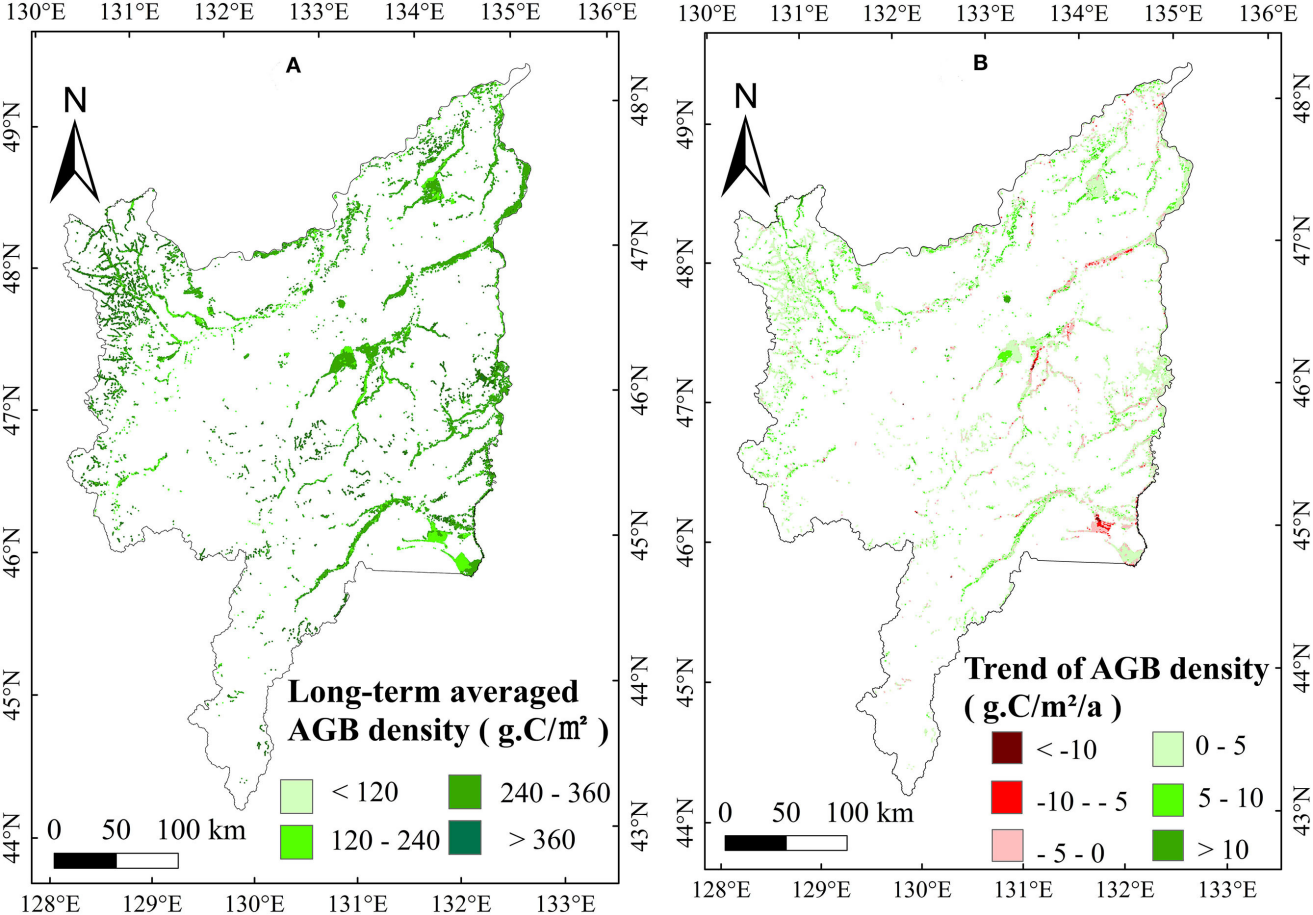
Distributions of long-term average AGB density (g·C/m^2^) **(A)** and change trends of AGB density (g·C/m^2^/a) **(B)** in the marsh of the Sanjiang Plain from 2000 to 2020.

Based on the NDVI_max_ and measured AGB dada, an optimal equation (power function, *R*^2^ = 0.86) was established for the Sanjiang Plain marsh (Figure 2A, Equation 5). We used the annual NDVI_max_ dataset to calculate the marsh AGB in Sanjiang Plain from 2000 to 2020. Results showed that the average marsh AGB density was approximately 282.05 g·C/m^2^ over the entire Sanjiang Plain from 2000 to 2020. The area of the Sanjiang Plain marsh was about 6.0 × 10^8^ m^2^, and thus the total marsh AGB of the whole Sanjiang Plain was about 0.17 Tg·C. Spatially, the long-term average marsh AGB density during 2000–2020 had a high value in the southeast and a low value in the northwest of the Sanjiang Plain (Figure 3A).


(5) $$\begin{array}{r} AGB\;density=643.57\times NDVI_{\text{max}}^{4.2474}\left(R^{2}=0.86\right) \end{array}$$

### Spatiotemporal Variation in Marsh AGB in the Sanjiang Plain

**Figure 4:**
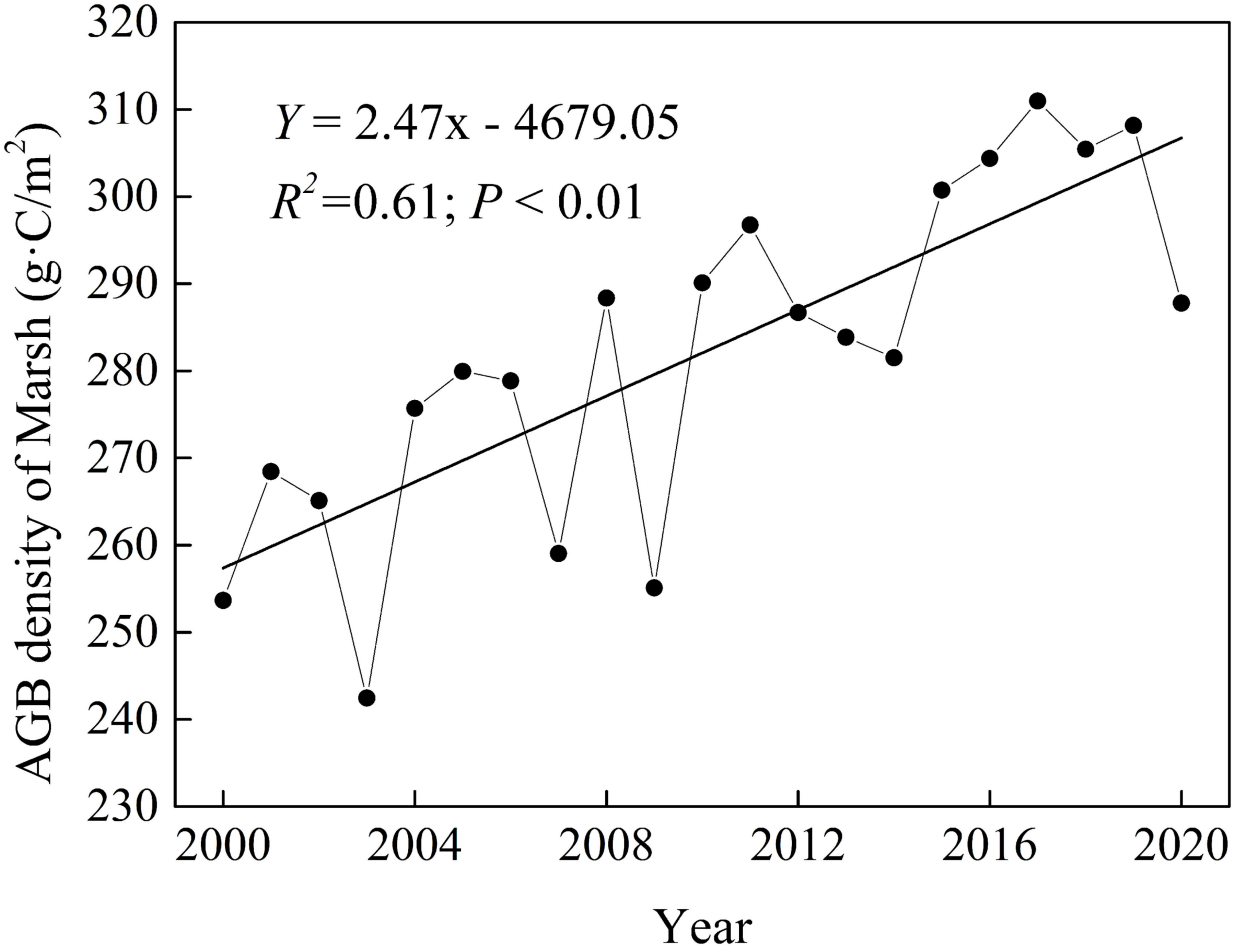
Temporal variation of AGB density in Sanjiang Plain's marshes during 2000–2020.

To study the temporal variation in marsh AGB density, we analyzed the time series of AGB density. Marsh AGB density had a significant (*p* < 0.05) upward trend (2.47 g·C/m^2^/a) over the entire Sanjiang Plain during 2000–2020 (Figure 4). The annual variation trend of the AGB density was spatially heterogeneous in the Sanjiang Plain marsh. The most significant increase in AGB density was observed in the north of the Sanjiang Plain, whereas decreased AGB density was mainly documented in the southeast (Figure 3B).

### Correlation Between Climate Factors and Marsh AGB in the Sanjiang Plain

**Figure 5:**
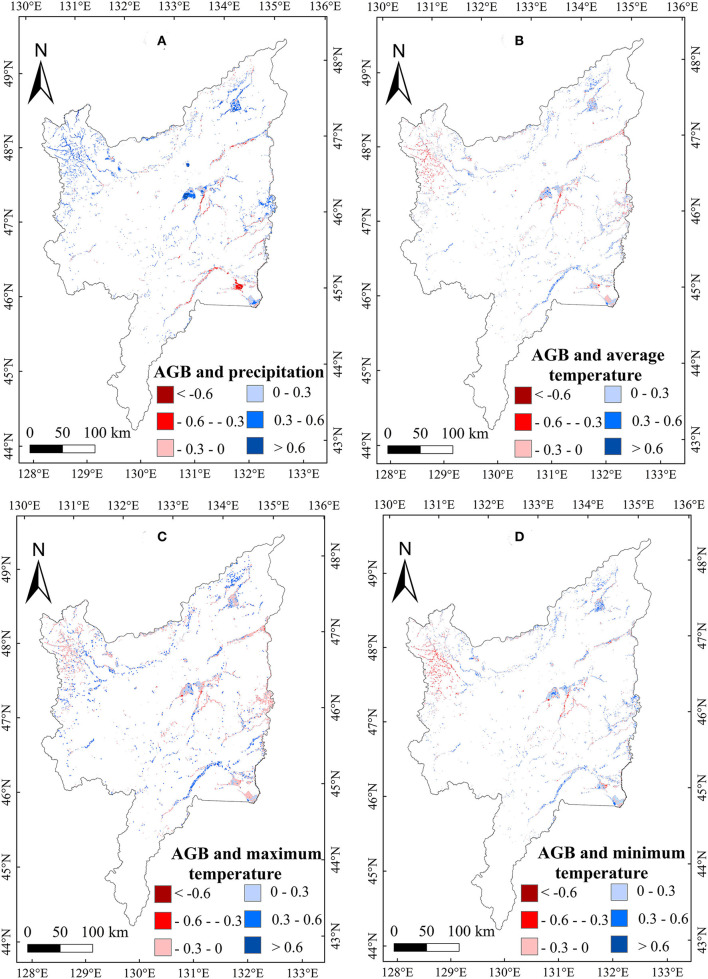
Spatial patterns in relationships of marsh AGB with annual precipitation **(A)**, T_mean_ **(B)**, T_max_ **(C)** and T_min_ **(D)** on the Sanjiang Plain marshes from 2000 to 2020.

**Figure 6:**
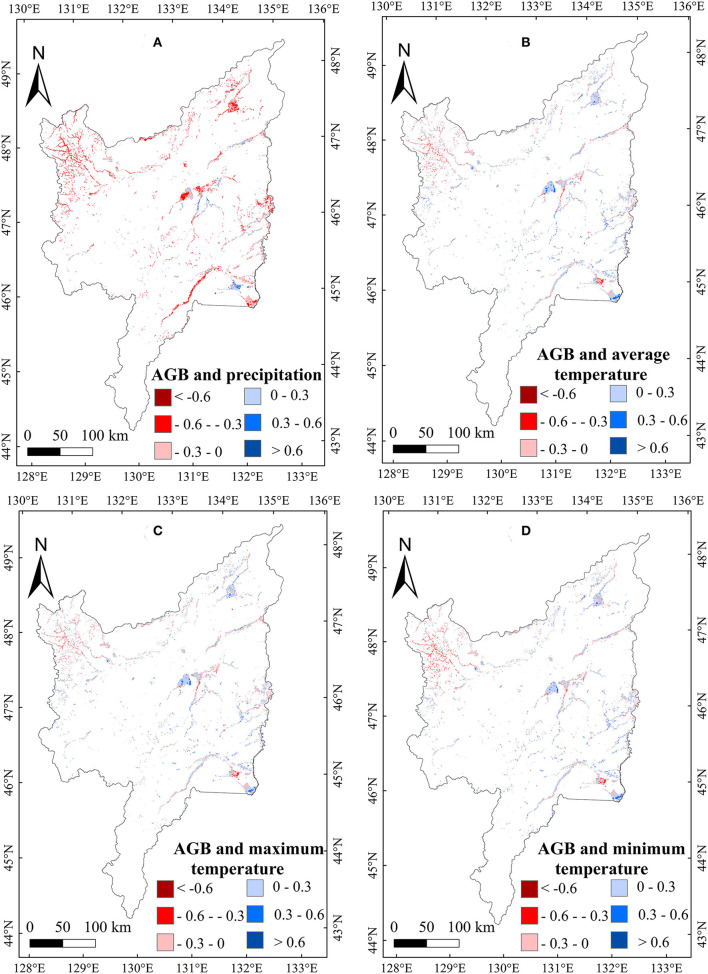
Spatial patterns in relationships of AGB with precipitation **(A)**, T_mean_ **(B)**, T_max_ **(C)**, and T_min_ **(D)** in January on the Sanjiang Plain marshes from 2000 to 2020.

**Figure 7:**
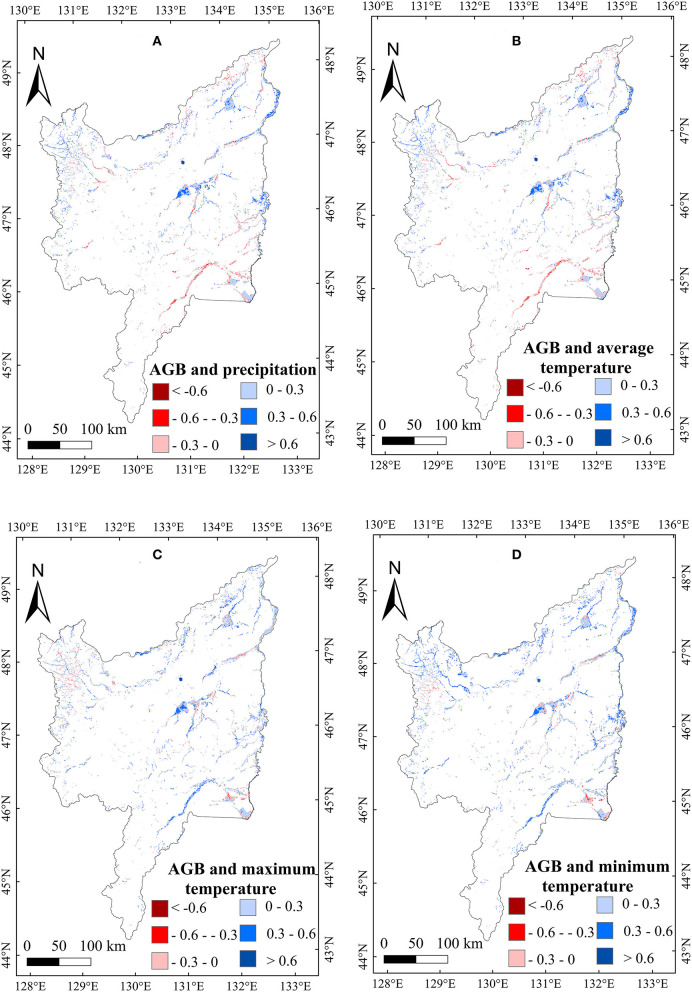
Spatial patterns in relationships of AGB with precipitation **(A)**, mean temperature (T_mean_) **(B)**, maximum temperature (T_max_) **(C)**, and minimum temperature (T_min_) **(D)** in July on the Sanjiang Plain marshes from 2000 to 2020.

To explore the relationship between meteorological variables and the AGB of marsh vegetation in the Sanjiang Plain, we calculated the correlations between meteorological variables and the AGB of marsh vegetation from 2000 to 2020. A weak positive correlation between the annual precipitation, T_min_, T_max_, and T_mean_, and AGB (Table 1) was found in the study area. Spatially, the correlation between AGB and annual precipitation was positive in most regions of the study area (Figure 5A). The positive correlation with annual temperature was mainly distributed east of the Sanjiang Plain (Figures 5B–D). For the seasonal climate effects, the correlation between precipitation in January and the vegetation AGB in the Sanjiang Plain marshes was significantly (*p* < 0.05) negative. By contrast, the correlations between AGB and mean temperature, minimum temperature, and maximum temperature in January were weakly positive (Table 1), and the positive correlations were mainly distributed in the middle of the Sanjiang Plain (Figure 6). In addition, correlations between the AGB and temperatures (such as T_mean_, T_max_, and T_min_) in July were significantly (*p* < 0.05) positive over the marshes of the Sanjiang Plain (Table 1), and the positive correlations were mainly concentrated in the north and middle of the Sanjiang Plain (Figure 7).

**Table 1:** Correlation coefficients between precipitation, mean temperature (T_mean_), maximum temperature (T_max_), minimum temperature (T_min_), and aboveground biomass (AGB) of marsh vegetation in the Sanjiang Plain from 2000 to 2020.

	**Precipitation**	**T_**mean**_**	**T_**max**_**	**T_**min**_**
Annual	0.394	0.149	0.321	0.178
January	−0.664^**^	0.156	0.091	0.162
February	−0.214	0.080	0.052	0.084
March	−0.041	0.149	0.176	0.170
April	0.086	−0.047	0.031	−0.223
May	0.394	−0.008	0.004	−0.065
June	0.134	0.144	0.140	0.174
July	0.132	0.620^**^	0.570^**^	0.599^**^
August	0.038	0.123	0.097	0.170
September	0.390	−0.236	−0.007	−0.279
October	0.101	−0.004	0.171	−0.220
November	0.050	−0.009	−0.029	−0.030
December	−0.202	0.112	0.054	0.115

** *means significantly at the levels of p < 0.01*.

## Discussion

### Verification of AGB Density Power Function

In this study, we established an optimal equation (the value of *R*^2^ was the largest in the equation than in other equations) between the NDVI_max_ and measured marsh AGB density (AGB density = 643.57 × NDVI${}_{\text{max}}^{4.247}$). Our findings were similar to those reported by Piao et al. (2004) and Wang et al. (2021), who reported that the AGB density was related to NDVI_max_, and a power function could fit them well. However, the power function is not the same in these studies. It may be because Piao et al. (2004) and Wang et al. (2021) studied grasslands in China and the marsh in the Qinghai-Tibet Plateau, respectively, whereas our study focused on the Sanjiang Plain marshes. Different research regions and objects may explain the reasons for varied equations. For evaluating the accuracy of estimated AGB density based on the relation equation, the estimated and observed AGB of the marsh were compared (Figure 2B). The RMSE and relative error in this power function were 43.50 and 13.89%, respectively. These results confirm our hypothesis that the marsh AGB density in the Sanjiang Plain can be accurately estimated by the NDVI_max_ in the Sanjiang Plain.

### Spatiotemporal Variation of AGB Density

Based on the constructed power function equation, this study calculated that the long-term average marsh vegetation AGB density in the Sanjiang Plain from 2000 to 2020 was approximately 282.05 g·C/m^2^. Our result was similar to that documented by Zhang et al. (2020), where the average AGB density of the Dongsheng wetland was approximately 299.70 g·C/m^2^. We found a larger AGB density than that of *Carex lasiocarpa* in the Sanjiang Plain marsh (209.16 g·C/m^2^) documented by Song et al. (2003). This may be because Song et al. (2003) studied *Carex lasiocarpa*, which has a relatively small biomass, while this study studied all the marsh vegetation in the Sanjiang Plain. Spatially, the long-term average marsh AGB density during 2000–2020 was high in the southeast and low in the northwest of the Sanjiang Plain (Figure 3A), which may be related to the high (low) vegetation cover in the warm southeast (cold northwest) of the Sanjiang Plain (Shen et al., 2019a). The largest declining trend in marsh AGB was found in the east of the Sanjiang Plain (Figure 3B), which could be accounted for by the marsh degeneration in these regions due to the interference from human activities (Yan et al., 2016).

### AGB Response to Climate Factors

**Figure 8:**
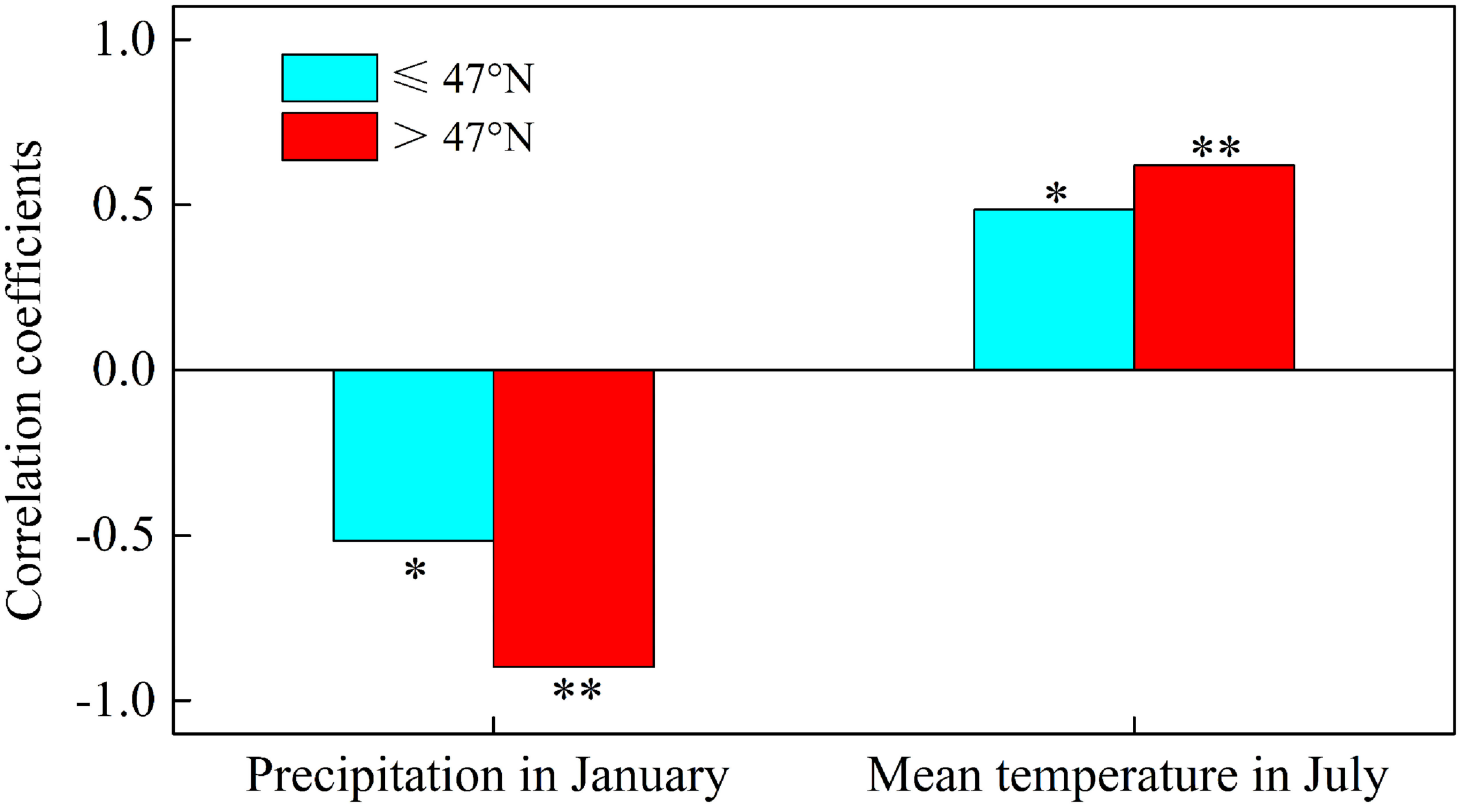
Correlation coefficients between marsh AGB and precipitation in January and mean temperature in July in two different regions of the Sanjiang Plain, south of 47°N ( ≤ 47°N) and north of 47°N (>47°N). ** and * mean significantly at the levels of *p* < 0.01 and *p* < 0.05, respectively.

Annual meteorological factors (temperature and precipitation) and AGB in the marshes of the Sanjiang Plain had a weakly positive relationship, which was similar to the findings of Guo et al. (2008) who found that annual precipitation and annual temperature were not the main factors affecting marsh plant growth in the Sanjiang Plain. The marsh AGB was negatively correlated with winter precipitation and the correlation with precipitation in January was significant (*p* < 0.05) (Table 1), indicating that precipitation in winter, especially in January, could inhibit marsh vegetation growth in the Sanjiang Plain. The increase in precipitation during winter can lead to cooling and insufficient heat accumulation, resulting in a retarded plant growth (Rajput et al., 1987; Yun et al., 2018). The AGB of the marsh vegetation in the Sanjiang Plain had a significant positive relationship with temperature in July. This finding was consistent with Yan et al. (2021) who found that there was a positive correlation between temperature and vegetation biomass in the Sanjiang Plain but was different from the finding of Wang et al. (2022a), who found that the increase of temperature in summer would reduce the biomass of marsh vegetation in the Songnen Plain. This may be because the climate of the Songnen Plain was the semi-arid climate, while the climate of the Sanjiang Plain was a temperate monsoon climate. Therefore, summer precipitation is not the main factor affecting marsh AGB in the Sanjiang Plain (Shen et al., 2019a). To confirm the hypothesis that temperature is the main reason affecting the AGB in the marsh of the Sanjiang Plain, we further calculated the correlation coefficients between marsh AGB and precipitation in January and mean temperature in July in two different regions of the Sanjiang Plain (south and north of 47°N). The results showed that the positive correlation of AGB with July temperature and the negative correlation of AGB with January precipitation was larger north of 47°N than south of 47°N (Figure 8). It confirms that the marsh AGB was mainly affected by temperature in the Sanjiang Plain, and the positive effects of temperature and negative effects of precipitation on marsh AGB were more obvious in much colder regions of the Sanjiang Plain.

Additionally, we found that warming at night-time and day-time had an asymmetric influence on marsh vegetation growth in the Sanjiang Plain, and warming at night had a greater effect on plant growth than warming during the day (Table 1). On the one hand, the temperature increase during the day promotes photosynthesis in plants and is conducive to the accumulation of organic matter (Xia et al., 2018), explaining that AGB has a positive correlation with T_max_ in July. On the other hand, the increase in T_min_ at night will bring a compensatory effect, which is beneficial to photosynthesis the next day (Hu and Kang, 2005; Xia et al., 2018). The over-compensation effect refers to the phenomenon that warming nighttime temperatures can promote photosynthesis and increase organic matter consumption during the next day to exceed the organic matter consumed due to enhanced respiration (Belsky, 1986; Peng et al., 2011; Chen et al., 2014). According to previous studies, when vegetation grows in sufficient nutrient and water environments, it has a high compensation effect and sometimes even shows an over-compensation effect (Belsky, 1986; Shen et al., 2021b). In July, owing to good hydrothermal conditions and sufficient nutrients (Wang et al., 2003), marsh vegetation in the Sanjian Plain has a strong photosynthetic capacity and it is easy to have an over-compensation effect (Wang et al., 2003). Therefore, the minimum temperature in July had a significant positive effect on the AGB in the marsh of the Sanjiang Plain.

**Figure 9:**
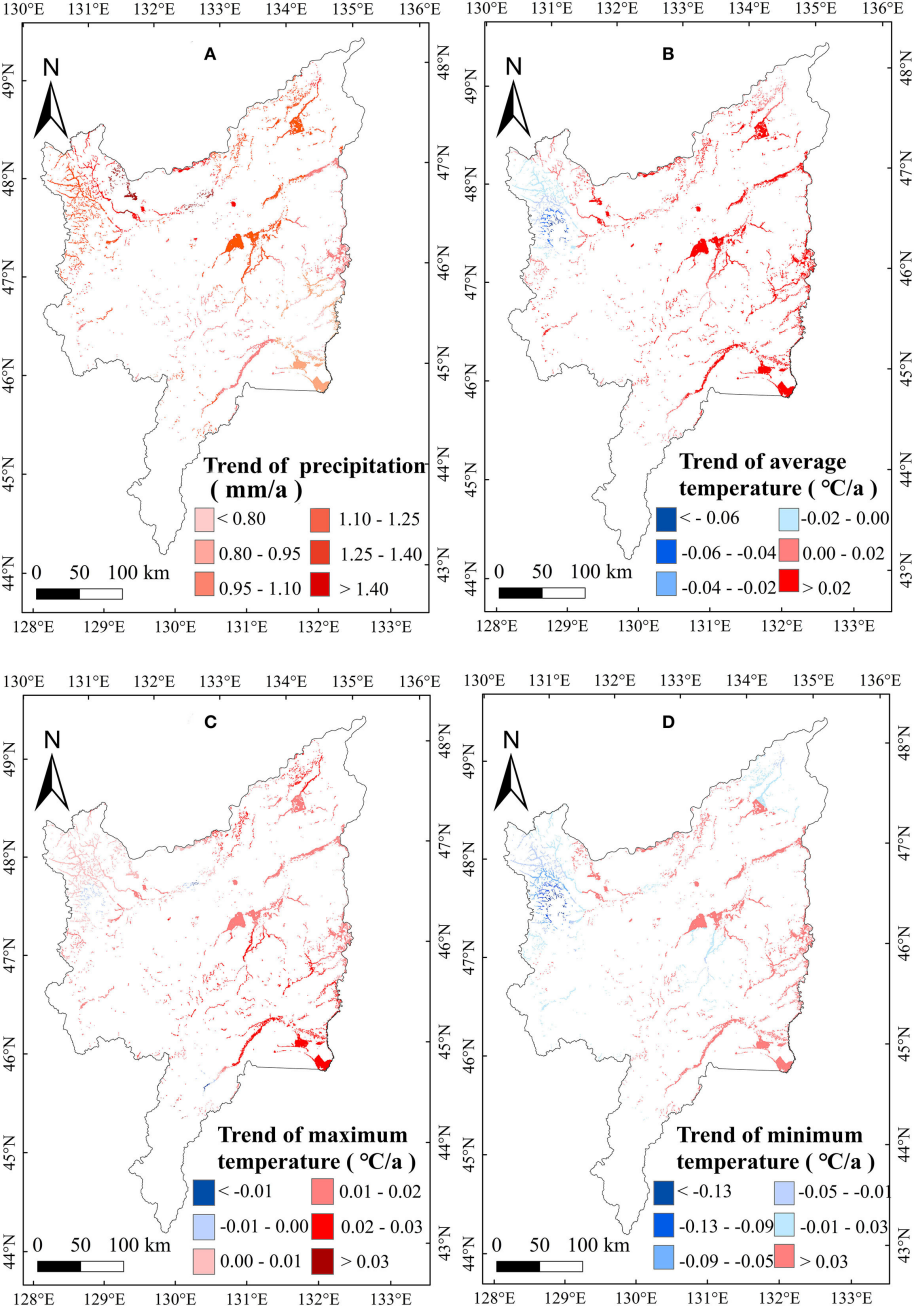
Change trends in annual precipitation (mm/a) **(A)**, T_mean_ (°C/a) **(B)**, T_max_ (°C/a) **(C)**, and T_min_ (°C/a) **(D)** in marshes of the Sanjiang Plain from 2000 to 2020.

**Figure 10:**
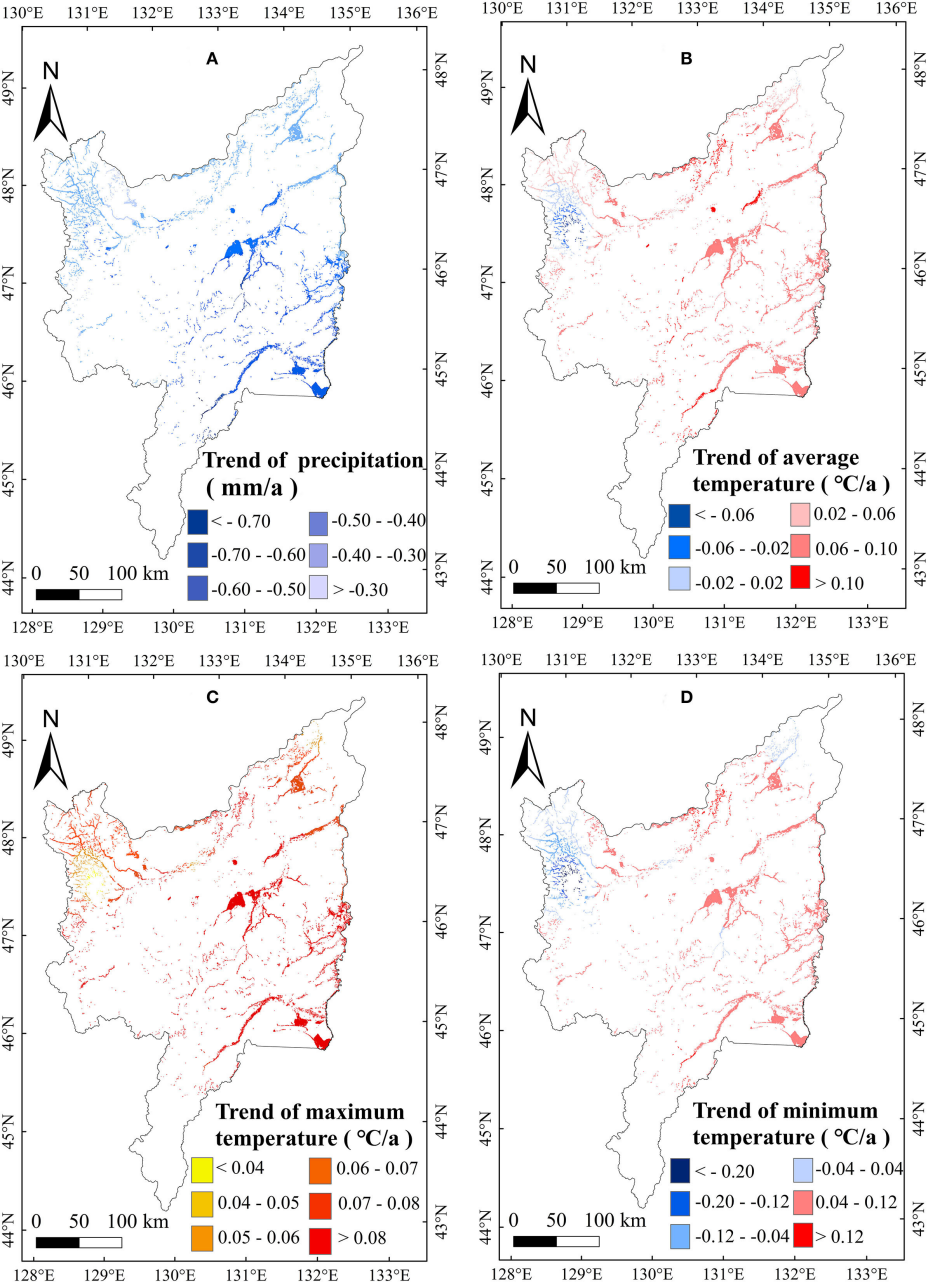
Change trends in precipitation (mm/a) **(A)**, T_mean_ (°C/a) **(B)**, T_max_ (°C/a) **(C)**, and T_min_ (°C/a) **(D)** in January in marshes of the Sanjiang Plain from 2000 to 2020.

**Figure 11:**
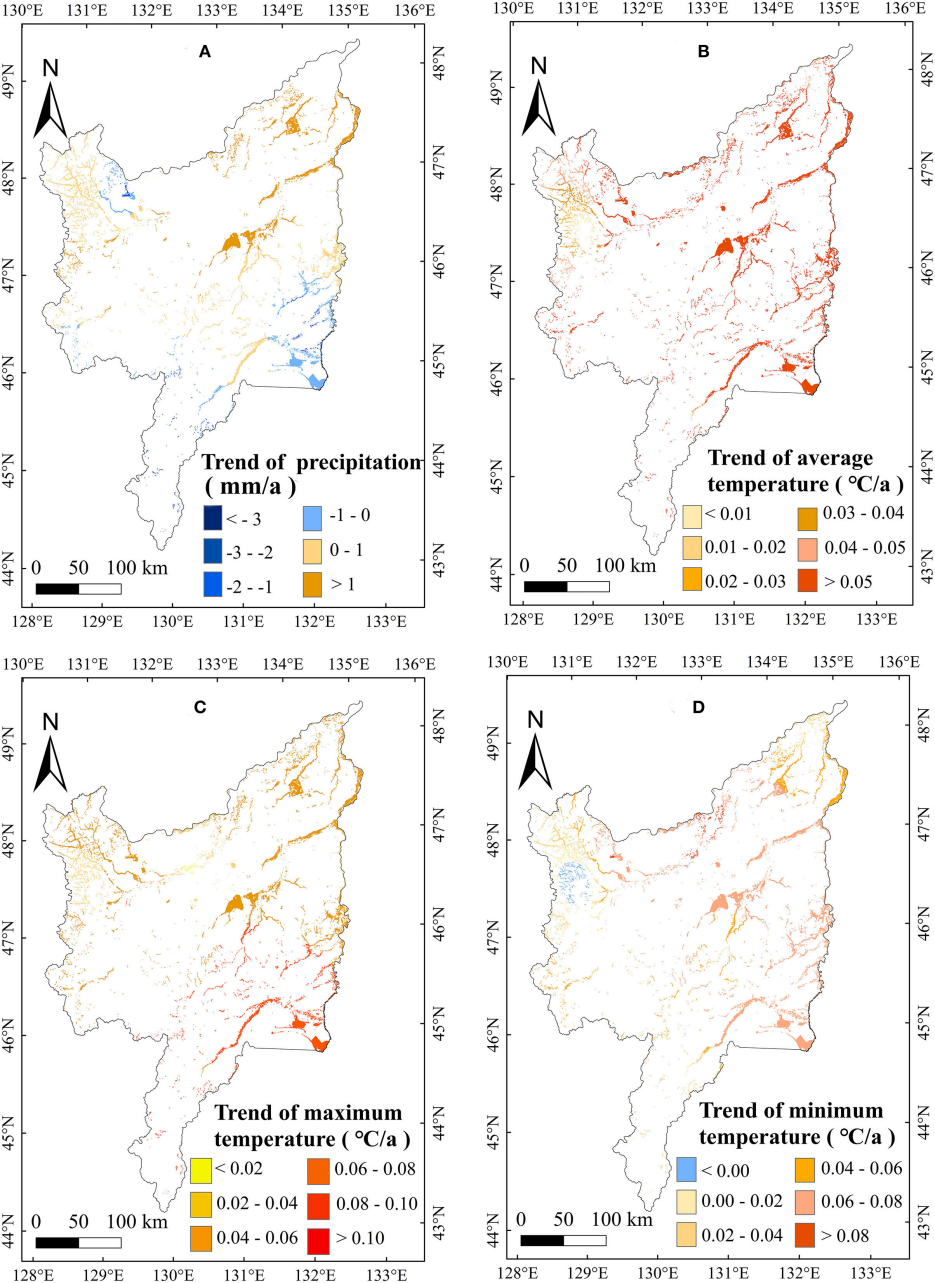
Change trends in precipitation (mm/a) **(A)**, T_mean_ (°C/a) **(B)**, T_max_ (°C/a) **(C)**, and T_min_ (°C/a) **(D)** in July in marshes of the Sanjiang Plain from 2000 to 2020.

To further explain the changes of marsh AGB, this study also analyzed the change trends in meteorological variables from 2000 to 2020 (Table 2). Annual precipitation increased significantly (1.14 mm/a) during the study period in the marsh of the Sanjiang Plain. Seasonally, the precipitation in January showed a significant decreasing trend (Table 2). Considering the significant negative correlation between the AGB and January precipitation (Table 1), the decrease in January precipitation (Table 2) may partly explain the increased AGB of marsh vegetation in the Sanjiang Plain. The annual temperatures (such as, T_min_, T_max_, and T_mean_) showed no significant increasing trends, but the temperature in June increased significantly (Table 2). Considering the significant positive correlation between the marsh vegetation AGB and the temperature in July (Table 1), the increase in temperature in July may partly explain the increase in marsh vegetation AGB at the Sanjiang Plain. The marsh AGB in the Sanjiang Plain showed an overall increasing trend from 2000 to 2020. The area with the largest increasing trend was concentrated in the center of the Sanjiang Plain (Figure 9). In the middle region of the Sanjiang Plain, precipitation decreased the most in January (Figure 10), and the temperature increased the most in July (Figure 11). According to the spatial correlation between the AGB and climate factors (Figures 6, 7), the decrease in precipitation in January and the increase in temperature in July may account for the increasing AGB of marsh vegetation in the middle of the Sanjiang Plain.

**Table 2:** Temporal trends in precipitation (mm/a), T_min_ (°C/a), T_max_ (°C/a), and T_mean_ (°C/a) in marshes of the Sanjiang Plain from 2000 to 2020.

	**Precipitation**	**T_**mean**_**	**T_**max**_**	**T_**min**_**
Annual	1.138**	0.021	0.046	0.027
January	−0.426*	0.084	0.090	0.071
February	0.005	0.032	0.034	0.017
March	−0.502	0.097	0.125	0.079
April	−0.464	0.023	0.073	−0.041
May	1.969	−0.002	0.000	0.060
June	0.585**	−0.342	−0.426	0.001
July	0.358	0.046*	0.053*	0.047*
August	4.339*	−0.020	−0.038	0.018
September	3.131*	−0.007	−0.040*	0.043
October	−0.200	0.017	0.041	−0.009
November	1.117*	0.028	0.001	0.036
December	0.161	0.038	0.016	0.039

^**^ and ^*^ mean significantly at the levels of p < 0.01and p < 0.05, respectively.

### Uncertainty

Although an effective method was provided for estimating the AGB density in the Sanjiang Plain marshes, this study may still have some limitations. First, the accuracy of NDVI data might be affected by factors, such as solar altitude angle, clouds, and atmosphere, which in turn could affect the results of this work. Second, the number of sampling points and meteorological stations was relatively limited, which may have caused some uncertainties in our results. Third, although this study extracted the unchanged marsh distribution in the Sanjiang Plain using two marsh distribution maps, it still could not rule out the impacts of anthropogenic activities on marshes in the study area. More marsh distribution data should be used to further analyze the marsh changes in the study area. Fourth, while numerous environmental factors affect AGB, we only analyzed the influences of temperature and precipitation on the marsh AGB of the Sanjiang Plain. Future research efforts should be directed toward understanding the influences of other factors on AGB in the marshes of the Sanjiang Plain.

## Conclusion

The results showed that marsh vegetation AGB density and NDVI_max_ had a good correlation, and the marsh AGB density in the Sanjiang Plain could be correctly calculated from a power function equation between NDVI_max_ and marsh AGB density (AGB density = 643.57 × NDVI${}_{\text{max}}^{4.2474}$). According to the function equation, the total marsh AGB of the entire Sanjiang Plain was approximately 0.17 Tg·C. The AGB density demonstrated a significant increasing trend of 2.47 g·C/m^2^/a in the Sanjiang Plain from 2000 to 2020, with an average AGB density of 282.05 g·C/m^2^. Spatially, the largest increasing trends of AGB density were located in the north of the Sanjiang Plain, whereas the decreasing trends were found in the southeast. Regarding climate effects, changes in annual precipitation and temperature had no significant impacts on marsh AGB in the Sanjiang Plain. The increasing precipitation in January could significantly decrease the marsh AGB while the warming temperature in July increased the marsh AGB in the Sanjiang Plain. This work proposed an effective approach for accurately estimating the AGB density of marsh vegetation in the Sanjiang Plain by using NDVI data and highlighted the importance of including monthly climate properties in models of marsh AGB dynamics in the marsh of the Sanjiang Plain.

## Data Availability Statement

The original contributions presented in the study are included in the article/supplementary material, further inquiries can be directed to the corresponding author.

## Author Contributions

XS coordinated the project. YL carried out the data analysis and wrote the manuscript. YW, JZ, RM, XL, and MJ contributed to modify the manuscript. All authors contributed to the article and approved the submitted version.

## Funding

This work was funded by the National Natural Science Foundation of China (41971065), Youth Innovation Promotion Association, CAS (2019235), the Natural Science Foundation of Jilin Province (20210101104JC), the Key Research Program of Frontier Sciences, CAS (ZDBS-LY-7019), and the Innovation Team Project of Northeast Institute of Geography and Agroecology, Chinese Academy of Sciences (2022CXTD02).

## Conflict of Interest

The authors declare that the research was conducted in the absence of any commercial or financial relationships that could be construed as a potential conflict of interest.

## Publisher's Note

All claims expressed in this article are solely those of the authors and do not necessarily represent those of their affiliated organizations, or those of the publisher, the editors and the reviewers. Any product that may be evaluated in this article, or claim that may be made by its manufacturer, is not guaranteed or endorsed by the publisher.
